# Validation of the Bangla WHO-5 Well-being Index

**DOI:** 10.1017/gmh.2021.26

**Published:** 2021-07-19

**Authors:** Md. Omar Faruk, Farzana Alam, Kamal Uddin Ahmed Chowdhury, Tanjir Rashid Soron

**Affiliations:** 1 Centre for Disability in Development (CDD), Dhaka, Bangladesh; 2 Dhaka University, Dhaka, Bangladesh; 3 Telepsychiatry Research and Innovation Network, Telepsychiatry Innovation Lab, Dhaka, Bangladesh; 4 University of Edinburgh, Edinburgh, UK

**Keywords:** Bangla, factor structure, reliability, validity, WHO-5 Well-being

## Abstract

**Background:**

Subjective wellbeing in terms of objective outcome can be useful to determine the level of progress in clinical practice as well as research studies in Bangladesh. Besides, cultural understanding of well-being for Bangladeshi population is also equally important to report. A valid Bangla version of the five-item WHO Well-being Index can be a suitable measure to achieve the purposes. Therefore, the present study aimed at validating the WHO-5 Well-being Index for general population in Bangladesh.

**Methods:**

After following the standard procedures for translation, back-translation, and committee translation, the initial Bangla version of the scale was developed and pretested. Based on the feedback during pretesting, a slight modification was made and the final version was developed. This final version was administered to 269 participants of different socioeconomic backgrounds to find out the reliability and validity of the scale from March 2019 to May 2019. The data analysis was conducted using SPSS 24.

**Results:**

The scale demonstrated acceptable internal consistency (*α* = 0.754) and test-retest reliability (*r* = 0.713), divergent validity (*r* = −0.443, *p* < 0.01 with the Bangla version of Perceived Stress Scale-10) and convergent validity (*r* = 0.542, *p* < 0.01 with the Bangla version of Warwick-Edinburgh Mental Well-Being Scale). The data also yielded one-factor structure for the scale in exploratory factor analysis explaining 38.68% of total variance. The factor-structure was further supported in the confirmatory factor analysis (χ^2^ = 295.852, χ^2^/df = 2.017, RMSEA = 0.062, CFI = 0.986, TLI = 0.964, and SRMR = 0.0255).

**Conclusion:**

The findings suggested the Bangla version of the WHO-5 Well-being Index is a psychometrically valid and reliable tool for general adult population in Bangladeshi when it comes to measuring subjective well-being both in clinical practice and research studies.

## Introduction

The concept of well-being has different connotations for different individuals, groups, and cultures (World Health Organization, 2017) making it difficult to reach a universally accepted definition of well-being. However, in general, well-being refers to the cognitive, emotional, and behavioral responses at a personal level. Psychological well-being can thus be interpreted in the sociocultural context of an individual and be seen in the form of a spectrum (World Health Organization, 2017). Such different connotations and the spectrum operating for an individual appear to be very subjective. Therefore, efforts to define well-being have led to a number of perspectives and measures. For instance, in a multi-disciplinary review, Dodge *et al*. (2012) argued that many attempts at expressing the nature of well-being have focused purely on dimensions of well-being, rather than on definition. Similarly, after reviewing 75 scientific articles with more than 100 different scales or questionnaires, Gill and Feinstein (1994) demonstrated that a clinimetric definition of subjective well-being was lacking. Thus, Gill and Feinstein (1994) advocated for the development of short global rating scales of subjective well-being reflecting a single dimension with high clinical face validity. The WHO-5 Well-being Index can be one such well-qualified measure for measuring subjective well-being.

With the translation into more than 30 languages, the five-item WHO Well-being Index is one of the most widely used questionnaires to assess subjective psychological well-being (Topp *et al*., 2015). In their systematic review, Topp *et al*. (2015) demonstrated four distinguished characteristics of the scale: the high clinimetric validity of the scale, the sensitivity of the scale in controlled clinical trials, potentiality to screen depression, and robustness of the scale across study fields. The WHO-5 Well-being Index has been reported as a pure generic instrument when it comes to measuring general well-being (Hall *et al*., 2011). The construct validity of the scale demonstrated a unidimensional scale with each item adding unique information about the level of well-being. The predictive validity of the scale has also been shown higher in many studies (Topp *et al*., 2015).

The WHO-5 Well-being Index has been used in a variety of areas along with research studies. The scale has been used in six controlled clinical trials as an outcome measure in the fields of psychiatry, oncology, endocrinology, otolaryngology, and depression to name a few. The use of WHO-5 Well-being Index in the diagnosis of depression has been demonstrated in numerous research studies and was shown as a highly sensitive measure with a sensitivity score of 0.93 and specificity of 0.83. Besides depression, the WHO-5 Well-being Index can be used in research studies to assess well-being over time or to compare well-being between groups. The scale has been used in many study fields with namely stress, neurology, suicidology, pain, health economics, and cardiology with endocrinology being the top field studied using the scale (Topp *et al*., 2015).

Validated in many countries across cultures as well as among targeted population, no such effort has yet been put in validating the WHO-5 Well-being Index for general population in Bangladesh. Therefore, the present study aimed to validate the five-item WHO Well-being Index into Bangla so that the index can be used for both clinical and research purposes.

There are few scales available to measure the well-being in Bangladesh. For example, designed for measuring children's well-being, Haque and Imran (2016) adapted the Stirling Children's Well-being Scale in Bangladeshi context putting further emphasis on establishing sensitivity and discriminant validity with the emergence of diagnostic features. In addition to that, Rahman and Imran (2013) also adapted the Warwick-Edinburgh Mental Well-Being Scale (WEMWBS) (Tennant *et al*., 2007) into Bangla but with no assessment of factor-structure. Huque and Begum (2005) developed a scale for measuring psychological well-being which lacks the determination of norms. The length (66 items) of the scale may create fatigue among responders. Against this backdrop, the need for validating the WHO-5 Well-being Index among general adult population with a view to determining the subjective feeling would be of paramount importance. The index is likely to contribute to the clinical practice as well as research studies in Bangladesh.

## Methods

### Study participants

Analysis of factor structure was one of the aims of the study. The rule of thumb for factor analysis considers a sample size of 200 as fair and 50 participants per factor is adequate (Wilson Van Voorhis and Morgan, 2007). The present study recruited 270 participants through purposive sampling in Dhaka city. Two hundred and sixty-nine participants (43.9% female and 55.8% male) aged 18–55 years (M = 24.66, s.d. = 7.45) were included in the study while one participant was excluded due to the unwillingness for using the responses in the study. Students as participants outnumbered (68.8%) the remaining participants that included businessperson, job holder, housewife, and unemployed participants (collectively 31.2%). Participants from all religious backgrounds comprised the main sample of the study. The demographic properties of 269 participants are presented in Table 1. Another 70 participants were also recruited for assessing test-retest reliability.

**Table 1.** Demographic properties of participants

Variable	*N* = 269 (%)
Sex	
Male	150 (55.8)
Female	118 (43.9)
Others	1 (0.4)
Occupation	
Student	185 (68.8)
Job Holder	63 (23.4)
Businessperson	16 (5.9)
Homemaker	4 (1.5)
Unemployed	1 (0.4)
Marital status	
Unmarried	220 (81.8)
Married	48 (17.8)
Widow	1(0.4)
Religion	
Islam	247 (91.8)
Hindu	13 (4.8)
Christian	5 (1.9)
Buddhist	1 (0.4)
Other	3 (1.1)
Education	
Up to primary	1 (0.4)
Primary to secondary	13 (4.8)
HSC	35 (13.0)
Up to Hons'	165 (61.3)
Masters' and Ph.D.	50 (18.9)
Missing	5 (1.9)

### Study procedure

Validating the Bangla version of the index was approved by the authority of the five-item WHO Well-being Index. The translation and back-translation procedures were carried out in accordance with the steps suggested by Gjersing *et al*. (2010). The original scale was given to five professionals including two psychiatrists, a language expert, and two clinical psychologists to translate it into Bangla language. An expert panel of five members comprising language experts and mental health professionals reviewed the translation process and checked their suitability. The expert panel reviewed the items in terms of their understandability and clarity for general people in Bangladesh. The reviewed items were then synthesized in a new version with all the issues addressed. The synthesized Bangla version was then given to another five independent professionals (two clinical psychologists, two language experts, and a psychiatrist) to blindly back-translate the items in the original language. The professionals involved in the back-translation were neither aware nor notified of the concepts under study. Three professionals including a language expert formed another expert committee to check the back-translation. The items were checked in terms of their semantic clarity, idiomatic, and conceptual equivalence. Two words were changed based on the consensus of the expert committee to make them more relevant for general people in Bangladeshi. The draft version of the scale was pretested on a sample of 35 participants. The pretesting aimed at the understanding of the questions being asked, whether the questions conveyed similar meaning to the participants and whether the participants were able to answer each question. The pretesting resulted in no further revision and the scale was, therefore, finalized. The final version of the scale was sent to the original author via email to check the accuracy of the resultant version. The scale was finalized for data collection with no objection from the author. The data were collected in Dhaka city by three psychology graduates trained by the second author prior to the data collection.

### Ethics

The research ethics committee of the Department of Clinical Psychology, University of Dhaka, Bangladesh approved the study (project ID #MS190602, approved on 10 July 2019). The ethical standards suggested for human participants were maintained throughout the research. Participants were provided with an informed consent form with their participation as voluntary. No monetary compensation for the participation was provided.

### Assessments

The following scales were used as instruments for the validation of the scale.

#### Perceived Stress Scale (PSS)

Originally developed by Cohen *et al*. (1983), the PSS 10 was used for its superior psychometric properties over the two other versions (PSS 14 and PSS 4). Moreover, PSS-10 demonstrated good internal consistency and test-retest reliability. Besides, it has been tested with diverse tools to determine the construct validity and resulted in moderate to strong correlation establishing it as a robust tool for measuring perceived stress (Mozumder, 2017). The Bangla translated version of PSS-10 was used for the present study. No published data on the reliability and validity were found for the Bangla version of the PSS-10. The Bangla version is available at Sheldon Cohen's Laboratory for the study of stress (Mozumder, 2017). The 10-item five-point Likert-type scale measures the degree to which an individual appraises his or her life as stressful. The scale combined both positive (4, 5, 7, 8) and negative items (1, 2, 3, 6, 9, 10). In case of negatively stated items, 0 signifies ‘never’, 1 ‘almost never’, 2 ‘sometimes’, 3 ‘fairly often’, and 4 ‘very often’ while the reverse can be seen in case of positive items.

#### Bangla version of Warwick-Edinburgh Mental Well-Being Scale (WEMWBS)

The WEMWBS was developed and validated in the UK by Tennant *et al*. (2007), and in 2013, the Bangla version of WEMWBS (Rahman and Imran, 2013) was validated. The 14 item five-point Likert-type scale measures mental well-being focusing entirely on positive aspects of mental health (Tennant *et al*., 2007). The scale is scored by summing responses to each item answered from ‘none of the time’ up to ‘all of the time’. The higher the score the better the mental well-being. The Bangla version of the scale revealed a score of Cronbach's *α* 0.77, split-half reliability 0.87, and test-retest reliability 0.72. Convergent validity was found to be −0.53 (with regards to the General Health Questionnaire-12) (Rahman and Imran, 2013).

#### Statistical methods

The demographic data were analyzed by descriptive statistics. Internal consistency reliability was assessed using Cronbach's *α* suggested by Nunnally (1967, 1978). Item analysis, test-retest reliability, divergent, and criterion validity were analyzed using Pearson correlations. Exploratory factor analysis was carried out with the maximum likelihood method. Multiple indices such as χ^2^, ratio of χ^2^ to df (χ^2^/df), root mean square error of approximation (RMSEA), and comparative fit index (CFI) were used to assess the adequacy of the model fit. The criteria for model fit were χ^2^ with *p* ⩾ 0.01, χ^2^/df ⩽ 2, RMSEA ⩽ 0.06, CFI ⩾ 0.95, SRMR ⩽ 0.08 (Mozumder, 2017). The relationship between the scores of one item and the scores of all other items was examined by inter-item correlation. Most of the researchers considered the average inter-item correlation should be between 0.20 and 0.40 suggesting homogeneity of all items in relation to the same content domain (Piedmont, 2014). In order to analyze the data, SPSS 24 (IBM Corp, 2016) and AMOS 18 (Arbuckle, 2009) were used.

## Results

The demographic properties of the participants are presented in Table 1.

The results included item analysis, reliability and validity assessment, and analysis of factor structure.

### Item analysis

Item analysis of the scale revealed a correlation ranging from *r* = 0.445 to *r* = 0.561, all significant at *p* < 0.01 indicating good internal consistency with very good items (Ebel and Fresbie, 1991, p. 232). Items no. 5 and 2 demonstrated a lower inter-item correlation compared to others on the scale. However, the correlation between the items falls within the suggested range (Piedmont, 2014). Cristobal *et al*. (2007) suggested that corrected item-total correlation values lower than 0.30 are not acceptable. The corrected item-total correlation of all items in the current scale demonstrated a value of above 0.30.

The inter-item correlation and item-total statistics are presented in Table 2.

**Table 2.** Inter-item correlation and item-total statistics of the Bangla version of the WHO-5 well-being index

	Inter-item correlation					Item-total statistics	
	WHO_1	WHO_2	WHO_3	WHO_4	WHO_5	Corrected item-total correlation	Cronbach's *α* if item deleted
WHO_1	1.000	0.372	0.430	0.322	0.422	0.526	0.709
WHO_2	0.372	1.000	0.468	0.460	0.267	0.537	0.706
WHO_3	0.430	0.468	1.000	0.449	0.283	0.561	0.697
WHO_4	0.322	0.460	0.449	1.000	0.355	0.542	0.703
WHO_5	0.422	0.267	0.283	0.355	1.000	0.445	0.738

### Convergent validity

Assessment of convergent validity was carried out using the Bangla validated version of WEMWBS (Rahman and Imran, 2013) and the Bangla version of the WHO-5 Well-being Index. The correlation between the two scales was found to be *r* = 0.542 (*p* < 0.01). Evidence suggests that a reliability coefficient ranging from 0.31 to 0.60 is adequate (Post, 2016). Convergent validity for the present scale was found adequate.

### Divergent validity

Divergent validity was assessed correlating the Bangla version of the WHO-5 Well-being Index with the Bangla version of PSS (Mozumder, 2017) as the study showed that stress can adversely impact subjective well-being (Ritchie *et al*., 2011). The correlation between the two scales was *r* = −0.443 (*p* < 0.01) demonstrating a low negative correlation (Mukaka, 2012).

### Internal consistency reliability

The Bangla five-item WHO Well-being Index yielded a score of Cronbach's *α* 0.754 demonstrating a moderate value (Shrout, 1998).

### Test-retest reliability

With a gap of 2 weeks, the scale was administered twice on a group comprising 70 participants. The test-retest reliability for the Bangla five-item WHO Well-being Index was *r* = 0.713 (*p* < 0.01) suggesting an acceptable result (Benson and Clark, 1982; Opacich, 1991).

### Exploratory factor analysis

Exploratory factor analysis with maximum likelihood was performed. No rotation method was used as the scale yielded a single factor. The suitability of factor analysis was determined by the score of Kaiser-Meyer-Olkin (0.775) and Bartlett's test of sphericity (χ^2^ = 293.093, *p* < 0.001). With an Eigenvalue of 1, the exploratory factor analysis yielded one factor explaining 38.68% of the total variance. The KMO measure of sampling adequacy was found to be acceptable comparing with the rule of thumb (Spicer, 2005).

All items had a factor loading >0.60 except item no. 5 (factor loading 0.500). Elimination of factor loadings with a value <0.32 is generally recommended (Tabachnick and Fidell, 2014). The scale illustrated factor loadings for each item above the recommended values.

The factor structure with loading score is presented in Table 3.

**Table 3.** Factor structure of the Bangla version of the WHO-5 Well-being Index

Sl.	Items	*F*
1	I have felt cheerful and in good spirits	0.602
2	I have felt calm and relaxed	0.656
3	I have felt active and vigorous	0.687
4	I woke up feeling fresh and rested	0.648
5	My daily life has been filled with things that interest me	0.500

Anti-image correlations, communalities, and correlation matrices were also investigated. Anti-image correlations refer to the reflection of the pairwise correlation remaining after partialing out the effects of other variables. It is suggested that the diagonals of the anti-image correlation matrix should be over 0.5 (Hauben *et al*., 2017). The anti-image correlation matrix ranges from 0.762 to 0.779 (see Table 3). Removal of an item with a communality score <0.2 is suggested (Child, 2006). Except for item no. 5, the remaining items demonstrated acceptable value (see Table 3). The correlation matrix demonstrated a low correlation between items no. 2 and 5 with a value of 0.267. However, the value was found to be acceptable as the inter-item correlation should be between 0.20 and 0.40 (Piedmont, 2014).

Anti-image correlation and communalities are presented in Table 4.

**Table 4.** Anti-image matrices and communalities of the Bangla version of the WHO-5 Well-being Index

	Anti-image matrices	Communalities	
Items No.	Anti-image correlation	Initial	Extraction
WHO_1	0.765^a^	0.304	0.362
WHO_2	0.787^a^	0.319	0.430
WHO_3	0.779^a^	0.342	0.472
WHO_4	0.777^a^	0.320	0.420
WHO_5	0.762^a^	0.233	0.250

a Measures of sampling adequacy (MSA)

### Confirmatory factor analysis

To test the goodness of fit for the one-factor structure, AMOS 18 (Arbuckle, 2009) was used to run the confirmatory factor analysis (CFA). It is recommended that χ^2^, RMSEA, CFI, and SRMR are the indices that at a minimum should be reported when testing the goodness of fit (Kline, 2008). However, a number of fit indices were taken into consideration for the present study such as χ^2^, χ^2^/df, RMSEA, CFI, TLI, and SRMR as a good fit of the model. The χ^2^, χ^2^/df, RMSEA, CFI, TLI, and SRMR were found to be 295.852 (*p* > 0.01), 2.017, 0.062, 0.986, 0.964, and 0.0255, respectively. RMSEA and χ^2^/df were found to be slightly higher than the values considered (Mozumder, 2017). Browne and Cudeck (1993) suggested an RMSEA value of 0.05 or less indicates a close fit. However, this value cannot be considered infallible due to its subjective judgment (Arbuckle, 2011) and often produces inappropriate decisions (Hu and Bentler, 1999). Therefore, an RMSEA value in the range of 0.05–0.08 has been considered a fair fit (Hu and Bentler, 1999; Hair *et al*., 2010; Awang, 2012). A value of χ^2^/df ranging from 2 to 1 or 3 to 1 suggests an acceptable fit (Carmines and McIver, 1981, p. 80) while others have recommended using ratios lower than 2 or as high as 5 when it comes to indicating a reasonable fit (Marsh and Hocevar, 1985). Evidence suggests that a value of CFI above 0.87 indicates a marginal fit (Dagnall *et al*., 2018). A CFI >0.95 indicates a relatively good model-data fit (Xia and Yang, 2018). TLI was also taken into consideration that produced a value of 0.964 indicating a good model fit (Xia and Yang, 2018).

**Fig. 1.**
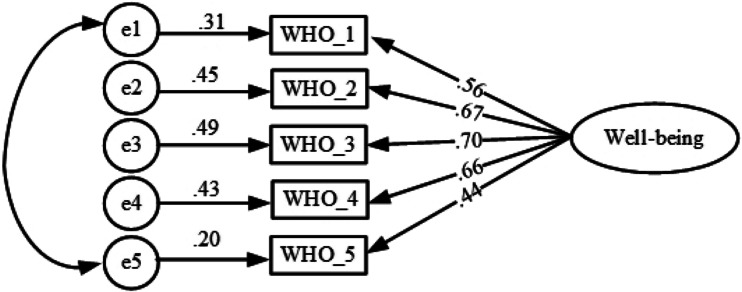
The one-factor structure of the Bangla version of the WHO-5 Well-being Index.

The one-factor initial analysis was found suggesting a correlation between error terms (items no. 1 and 5). Putting the error terms into the model, a better fit was found. The findings are presented in Table 5 and Fig. 1.

**Table 5.** Goodness of fit indices for one-factor model of the Bangla version of the WHO-5 Well-being Index

	χ^2^	df	*p*	χ^2^/df	RMSEA (CI)	CFI	SRMR
Original model	295.852	4	0.001	3.950	0.105 (0.059-0.155)	0.948	0.0446
Modified model	295.852	4	0.089	2.017	0.062 (0.000-0.123)	0.986	0.0255

## Discussion

The concept of well-being is subject to change depending on a given cultural context. For example, personality patterns, life circumstances, and other cultural variables may shape the meaning of well-being (Diener *et al*., 2003; Tov and Diener, 2007). Therefore, any psychometric tool measuring well-being should be in relation to the particular context. Hence the present study was taken into consideration. Suggested measures were followed during the translation processes to ensure cultural representation.

The scale was administered on a sample of 269 participants representing the general population. The scale demonstrated good internal consistency (Ebel and Fresbie, 1991, p. 232) for all items with scores ranging from 0.445 to 0.561. The inter-item correlation and the item-total statistics suggested further consistency of the scale.

Exploratory factor analysis (EFA) of the items yielded a one-factor structure for the Bangla version of the WHO-5 Well-being Index suggesting a consistent finding with the previous studies (Awata *et al*., 2007; De Wit *et al*., 2007; De Souza and Hidalgo, 2011; Hajos *et al*., 2013; Mortazavi *et al*., 2015; Chongwo *et al*., 2018; Dadfar *et al*., 2018). Of the five items, the first four items showed a loading value above 0.60 while the remaining items showed a value of 0.500. Anti-image matrices and communalities were found to be acceptable (see Table 4).

CFA was used to test further the one-factor model obtained in the EFA. The study has shown that cross-cultural validation involving different data sets results in a lack of correspondence between EFA and CFA (Van Prooijen and van der Kloot, 2001). To rule out the problem, CFA was carried out on the same data set. Error items (items 1 and 5) were correlated in the same factor (see Fig. 1), as correlating error terms within factors are found to be a common phenomenon (Gerbing and Anderson, 1984). The resulting model demonstrated a good fit when multiple indices (χ^2^, χ^2^/df, RMSEA, CFI, TLI, and SRMR) were considered to avoid inappropriate findings (Lai and Green, 2016). The model fit suggested considerable support for the Bangla version of the WHO-5 Well-being Index.

The scale displayed a value of Cronbach's *α* 0.754 that falls within the ranges (0.70–0.95) recommended in general (Shrout, 1998; Tavakol and Dennick, 2011). The scale was administered twice on a group of 70 participants demonstrating an acceptable test-retest reliability score of 0.713 (Benson and Clark, 1982; Opacich, 1991). Analysis of convergent validity revealed an adequate coefficient value (*r* = 0.542) (Post, 2016) when the Bangla version of the index was correlated with the Bangla version of WEMWBS (Rahman and Imran, 2013). A moderate correlation coefficient (*r* = −0.443) (Mukaka, 2012) value between the index and the Bangla version of the PSS (Mozumder, 2017) ensured the divergent validity.

The scale is the first of its kind to assess factor structures of well-being measures in Bangladesh. The psychometric measures validated or developed in Bangladesh did not assess the factor structure of the items or targeted only for children. Besides, the scale developed to measure psychological well-being contained a large number of items (Huque and Begum, 2005). Studies demonstrated that psychometric measures short or medium in length can be useful in terms of performance compared to longer scales (Kearns *et al*., 1982; Zigmond and Snaith, 1983; El-Rufie and Absood, 1995). The Bangla version of the WHO-5 Well-being Index can be a suitable tool to minimize the shortcomings mentioned above. The scale has been used as an outcome indicator in many research studies such as suicidology, alcohol abuse, diabetes, stroke, cancer, sleep problems, personality disorder, grief, and quality of life across the world (Topp *et al*., 2015). The Bangla version of the scale can also be used to assess the level of well-being in both clinical as well as research studies.

The study acknowledges a few limitations. As the study was conducted based on the Clinical Psychotherapy Unit of the University of Dhaka, an abundance of student participants was observed that skewed from the general adult population alongside other attributes. However, as the students came here from different backgrounds and every district of the country, this sample may be considered as a good representation of the country and Bangladeshi culture. Moreover, the scale used very simple and culturally neutral language, so it might have less influence on education and culture. The level of understanding in the Bangladeshi population of different backgrounds and ages was assessed during the pretesting. Determining the cut-off score in order for enabling the index as a screening tool was evident in a number of studies (Topp *et al*., 2015). However, as the present study attempted to validate the index among the general population without a clinical diagnosis, the cut-off score could not be determined marking it another limitation of the study. With a large sample size, it is also suggested to determine the diagnostic properties of the scale in future studies.

Despite several advantages such as less time-intensive, easy, and cheaper, non-probability sampling (purposive sampling for the study) can be subjected to selection bias (Forster, 2001; Galloway, 2005). Furthermore, self-reporting scales can produce inaccurate results led by social desirability bias (Kunda and Spencer, 2003). Therefore, employing probability sampling with measures that reduce biases of all forms is recommended in future studies.

## Conclusion

The Bangla version of the WHO-5 Well-being Index demonstrated reliable and valid psychometric properties in the Bangladesh context. It takes about 3–4 minutes to administer. This tool can help in tapping into the subjective well-being of the general population both in clinical as well as research studies in Bangladesh.
